# Genome-Wide Association Study of Body Measurement Traits at Birth in Cattle

**DOI:** 10.3390/ani16193133

**Published:** 2026-10-06

**Authors:** Xinlu Wang, Weiguo Ju, Xiaoming Ma, Min Chu, Yongfu La, Xian Guo, Ping Yan, Bo Yang, Chunnian Liang

**Affiliations:** 1 Key Laboratory of Animal Genetics and Breeding on Tibetan Plateau, Ministry of Agriculture and Rural Affairs, Lanzhou 730050, China; wangxinlu1996@163.com (X.W.); maxiaoming@caas.cn (X.M.); chumin@caas.cn (M.C.); layongfu@caas.cn (Y.L.); guoxian@caas.cn (X.G.); yanping@caas.cn (P.Y.); 2 Key Laboratory of Yak Breeding of Gansu Province, Lanzhou Institute of Husbandry and Pharmaceutical Sciences, Chinese Academy of Agricultural Sciences, Lanzhou 730050, China; 3 Zhangye Livestock Breeding and Improvement Workstation, Zhangye 734000, China; m13689329007@163.com

**Keywords:** cattle, genome-wide association study, body measurement traits, candidate genes

## Abstract

Hexi cattle are an indigenous beef population developed through multigenerational crossing of Simmental and Mongolian cattle, exhibiting exceptional adaptability to arid and semi-arid environments alongside robust growth performance. To investigate the genetic architecture underlying early growth traits, we conducted a GWAS for body weight (BW), withers height (WH), body length (BL), and heart girth (HG) in a cohort of 264 Hexi cattle (125 males, 139 females). Phenotypic analyses showed that male calves had significantly higher values for all traits relative to female calves (*p* < 0.05), confirming the presence of sexual dimorphism. The genomic inflation factors (λ = 0.997–1.016) indicated that population structure was effectively controlled. In total, 11 functional candidate genes were identified under the genome-wide significant threshold of *p* < 1.0 × 10^−6^. Specifically, *SLC44A1* associated with BW; *PTGS2*, *POP1*, and *ERC2* with WH; *TIAM1*, *PROK1*, *SMOC2*, and *RFFL* with BL; and *C3orf80*, *PROKR1*, and *FBXO32* with HG. These results not only advance our understanding of the genetic regulation of early development in Hexi cattle but also establish a foundation for implementing marker-assisted and genomic selection in this population.

## Simple Summary

Hexi cattle are an important indigenous beef population in China, known for their strong adaptability to dry environments and excellent growth performance. However, the genetic factors influencing their early growth traits remain largely unclear. In this study, we analyzed the genomes of 264 Hexi calves to identify genes associated with body weight and three body size traits at birth. We found that male calves were significantly larger than females across all measured traits. Using a genome-wide association approach, we identified 11 candidate genes that may play key roles in early growth and development. These genes are promising targets for future breeding programs, as they could be used to select calves with superior growth potential from an early age. Our findings provide valuable genetic resources that can help accelerate genetic improvement and enhance breeding efficiency in Hexi cattle populations.

## 1. Introduction

Indigenous livestock breeds constitute irreplaceable genetic reservoirs molded by long-term natural and artificial selection under diverse ecological and rearing environments [1]. Systematic crossbreeding of indigenous breeds with improved exotic breeds is a globally recognized breeding strategy to improve production performance while retaining the adaptive advantages of local livestock, and this approach has been validated in extensive large-scale breeding practices across the world [2]. A paradigmatic example of this strategy is the Hexi cattle, a crossbred population in Northwest China developed over four decades of systematic crossbreeding and selection between indigenous Mongolian cattle and Simmental; ADMIXTURE analysis of whole-genome resequencing data revealed that its genome comprises approximately 57% Simmental and 18% Mongolian ancestry [3]. This breeding program has successfully combined the robust adaptability of Mongolian cattle with the superior growth potential of Simmental, resulting in a population with exceptional production performance. Its unique admixed genomic architecture renders Hexi cattle an ideal model for dissecting the polygenic basis of artificial selection and elucidating the molecular mechanisms underlying phenotypic divergence. Nevertheless, the genomic architecture underlying these favorable phenotypes—specifically, the identification of trait-associated variants and the estimation of their genetic effects—has not yet been systematically characterized in this admixed population via high-depth whole-genome resequencing genome-wide association study (GWAS).

Dissecting the genetic architecture of economically important traits, including growth performance, carcass quality and reproductive efficiency, constitutes a core priority in modern beef cattle genetic improvement [4,5,6]. These complex polygenic quantitative traits are susceptible to environmental modulation and present abundant phenotypic variation among individuals [7]. Restricted by insufficient clarification of the underlying molecular regulatory networks, conventional phenotypic selection cannot achieve accurate and targeted genetic improvement, and cannot support the implementation of efficient genomic selection schemes [8]. The rapid advancement of high-throughput genomic technologies, particularly the widespread application of GWAS, has greatly facilitated the genetic dissection of complex economic traits in livestock [9]. GWAS uses genome-wide single nucleotide polymorphisms (SNPs) as molecular markers and exploits population linkage disequilibrium (LD) to identify genetic variants associated with target phenotypes [10]. To date, GWAS has been widely implemented in beef cattle studies and successfully uncovered numerous QTLs and candidate genes modulating growth efficiency, carcass quality, and fertility [11,12].

Cumulative GWAS analyses across multiple cattle breeds have pinpointed core genomic regions governing bovine growth and skeletal development. Notably, a pleiotropic QTL on bovine chromosome 6, with *LCORL* and *NCAPG* as prime candidate genes, has been repeatedly associated with body height and weight traits across Simmental and Belgian Blue populations [13]. Similarly, multi-breed meta-analyses have firmly established the association between variants of *HMGA2* and skeletal growth traits, including body height and chest circumference [14]. Further GWAS screening in Belgian Blue cattle has uncovered novel missense variants in *EZH2*, *PAPPA2*, and *ADAM12*, all of which exert key functions in regulating bovine growth [15]. In Charolais cattle, SNPs within the *ACOX1* gene have been shown to coordinately regulate growth performance and backfat thickness [16]. A previous GWAS based on a 50K SNP array in 254 Qinchuan cattle identified 250 significant SNPs annotated to 37 functional candidate genes, including *ADAMTS17*, *ALDH1A3*, and *MEF2A*, all of which participate in bovine growth and development [17]. Collectively, these findings validate that GWAS is a powerful tool for dissecting the genetic architecture of complex growth traits in cattle. Despite abundant GWAS efforts focusing on bovine body measurement traits, few systematic studies have integrated high-depth whole-genome resequencing to unravel the genetic regulatory mechanisms governing body measurement traits in Hexi crossbred cattle, and the population-specific functional genes and causal variants remain uncharacterized.

In this study, a GWAS was performed using whole-genome resequencing data from Hexi cattle to systematically detect candidate genes and genomic regions significantly associated with four growth-related body measurement traits, namely body weight, withers height, body length, and heart girth. The findings are expected to contribute to the elucidation of the polygenic regulatory mechanisms governing these traits, thereby furnishing a theoretical basis for precision breeding and genetic improvement in Hexi cattle, while simultaneously offering valuable molecular marker resources to expedite genetic progress in this population.

## 2. Materials and Methods

### 2.1. Animals, Phenotype Measurement, and Blood Collection

The experimental animals consisted of 264 clinically healthy Hexi cattle (125 males and 139 females) obtained from three core breeding farms in Zhangye, Gansu, China. All individuals were born within the same season, and all phenotypic measurements were performed strictly on the day of birth. All experimental animals were raised under consistent and unified breeding environment and feeding management conditions to eliminate the confounding effects of birth season, birth year, and other heterogeneous environmental factors on phenotypic traits. A consistent feeding and management scheme was adopted for all experimental calves. Trained technicians measured body weight (BW), along with three body size traits, including withers height (WH), body length (BL), and heart girth (HG), in the morning under fasting conditions. The measurement procedure strictly followed GB/T 43838-2024 (*Technical Specifications for Beef Cattle Production Performance Measurement*) [18]. Blood was obtained from the jugular vein using EDTA-K_2_ anticoagulant vacuum tubes, and about 5 mL of whole blood was taken from each calf. After collection, tubes were gently inverted 5 to 8 times for homogeneous mixing, and samples were preserved at −20 °C awaiting genomic DNA isolation.

### 2.2. DNA Extraction and Sequencing

Genomic DNA was purified from peripheral whole blood using a Tiangen extraction kit (Tiangen Biotech, Beijing, China), strictly following the protocols supplied by the manufacturer. Agarose gel electrophoresis (1%) was performed to verify DNA integrity, and a NanoDrop 2000 spectrophotometer (Thermo Fisher Scientific, Waltham, MA, USA) was employed to determine DNA concentration. Specimens satisfying all quality criteria were forwarded to Smart Biotech (Qingdao, China) for whole-genome resequencing.

### 2.3. Data Quality Control and Population Genetic Structure Analysis

In the present study, whole-genome resequencing was performed on 264 Hexi cattle. Raw read quality assessment and quality control (QC) were performed at the initial stage of sequencing data analysis. Briefly, FastQC was used to evaluate the basic quality metrics of raw reads, including total read counts, GC content, and Q20/Q30 values. Then, fastp was applied to trim adapter sequences, remove low-quality reads and filter reads with excessive N bases. After QC, the obtained clean reads were used for subsequent read mapping and downstream analyses. Clean sequencing reads were aligned to the bovine reference genome ARS-UCD1.2 (BosTau9, accession GCF_002263795.1) using BWA-MEM (v0.7.17) [19]. The resulting SAM files were converted to coordinate-sorted BAM files using SAMtools (v1.9) [20]. The sequencing depth exceeded 20× for every sample. SNP calling was performed using GATK (v4.2.6.1) with default parameters [21]. Functional annotation of the variants was performed using SnpEff (v5.0). Genotype quality control (QC) was then performed using PLINK (v1.90) [22]. Only autosomal SNPs that satisfied the following quality thresholds were retained for downstream analyses: genotype missing rate ≤ 0.1, minor allele frequency (MAF) > 0.01, and Hardy–Weinberg equilibrium *p* > 1 × 10^−6^. Variants located on the mitochondrial genome, sex chromosomes, or those with ambiguous genomic positions were excluded. After the above filtering steps, a final set of 22,613,679 high-quality autosomal SNPs was retained for downstream analyses.

Principal component analysis (PCA) of the Hexi cattle population was performed using PLINK (v1.90). Prior to PCA, LD pruning was performed using a 50 SNP sliding window with a 5 SNP step, removing SNPs with pairwise r^2^ > 0.2 to reduce bias from correlated markers; only the pruned SNP set was used for PCA. The first five principal components (PCs) were extracted, and the top two PCs were retained for visualization as they captured the largest proportion of the genetic variance. A two-dimensional PCA scatter plot was generated using the R package ggplot2 (v3.5.2) to visualize population clustering patterns. A genomic kinship matrix (K-matrix, also defined as GRM) was constructed based on high-quality filtered autosomal SNPs to evaluate pairwise genomic relatedness among individuals. Specifically, the K-matrix adopted for the FarmCPU GWAS model was calculated via the MVP. K. VanRaden function in the R package rMVP was used, following VanRaden’s Method 1 [23] as described by Yin et al. [24]. This precomputed K-matrix was used as the covariance matrix for random genetic effects in the FarmCPU association analysis. Genome-wide linkage disequilibrium (LD) decay was assessed using PopLDdecay (v3.42) [25]. Pairwise SNP r^2^ values were calculated within 1 Mb sliding windows, and the average r^2^ for each distance interval was plotted against the physical distance to generate the LD decay curve.

### 2.4. Genome-Wide Association Study

In this study, GWAS analyses were performed using the Fixed and Random Model Circulating Probability Unification (FarmCPU) model implemented in the R package rMVP [24,26]. To adjust for population stratification, we included farm, sex, and the first five principal components (PCs) obtained from genome-wide SNP markers in the model as fixed predictors. A kinship matrix was added as a random component to account for polygenic genetic background and cryptic relatedness across individuals. The iterative FarmCPU framework is described as follows:

y = Xα + Zβ + Wμ + e(1)

where y denotes the vector of individual phenotypic observations; X represents the design matrix for fixed factors, and α refers to the vector of estimated fixed effects encompassing farm environment, sex, and the top five principal components (PCs) for population stratification correction; Z is the genotype matrix of tested SNPs, and β indicates the vector of additive SNP effects estimated; W is the incidence matrix accounting for polygenic background, and μ is the vector of random polygenic effects following a multivariate normal distribution μ ∼ N (0, Kσ), where K is the kinship matrix and σ denotes polygenic variance; e is the vector of independent residual errors following e ∼ N (0, Iσ), in which I is the identity matrix and σ represents residual variance.

To reduce false-positive signals induced by multiple hypothesis testing, a genome-wide suggestive threshold line of *p* = 1 × 10^−6^ was set for Manhattan plots in this work, which is consistent with previous studies [3]. We estimated the genomic inflation factor (λ) for every trait to examine how population stratification shapes GWAS outcomes, and quantile-quantile (Q-Q) plots were constructed within R (v4.3.1).

### 2.5. Genotype Extraction and Association Analysis

Four SNPs were randomly selected from the genome-wide significant loci for each trait, with the requirement that each selected SNP possessed three distinct genotype groups for phenotypic comparison. SNP genotypes were extracted from the whole-genome sequencing data of the 264 Hexi cattle using bcftools (v1.9). Before genotype-effect comparisons, genotype groups with fewer than three individuals were excluded. Only groups with at least three animals were retained for subsequent statistical tests to ensure statistical reliability. For each trait, one locus was randomly selected from the candidate SNPs for further validation analysis. The online tool GDICALL (http://www.msrcall.com/gdicall.aspx, accessed 26 August 2026) was utilized to calculate homozygosity (Ho), heterozygosity (He), polymorphism information content (PIC), genotype frequencies, and allele frequencies, and to determine the *p*-values of the chi-square test and Hardy–Weinberg equilibrium test. To preliminarily explore the genotypic effects on each trait, a one-way ANOVA was conducted using SPSS Statistics 25 (IBM, New York, NY, USA), with genotype as the fixed factor and the respective body measurement trait as the dependent variable. When significant genotypic effects were detected, Tukey’s HSD test was used for post hoc pairwise comparisons among genotype groups. All results are presented as mean ± standard deviation (SD).

### 2.6. Phenotypic Variance Explained (PVE) Calculation

The proportion of phenotypic variance explained (PVE) by each significant SNP was estimated under an additive genetic model using the following formula [27]:

(2) $$\mathrm{PVE}=\frac{2\mathrm{MAF}X\left(1-\mathrm{MAF}\right)\beta^{2}}{2\mathrm{MAF}\left(1-\mathrm{MAF}\right)\beta^{2}+N\times {\mathrm{SE}}^{2}}$$

where β is the estimated SNP effect, SE represents the standard error of the SNP effect, MAF is the minor-allele frequency, and N = 264 is the total sample size in this study. Given that multiple significant SNPs within the same genomic region are in strong linkage disequilibrium and are not independent quantitative trait loci, we did not calculate a simple cumulative PVE across candidate genes to prevent upward bias.

## 3. Results

### 3.1. Sexual Differences in Body Weight and Measurement Traits

Descriptive statistics presented in Table 1 reveal significant sexual differences in body weight and body measurement traits among newborn Hexi cattle. All measured traits including body weight (BW), withers height (WH), body length (BL), and heart girth (HG) were significantly greater in males than in females (*p* < 0.05), indicating a consistent developmental advantage in overall body measurement traits for male calves. Regarding variability, BW exhibited the highest coefficient of variation (CV)—7.70% in males and 8.97% in females—suggesting relatively greater dispersion in this trait. In contrast, WH, BL, and HG showed markedly lower CVs (4.14%, 5.83%, and 5.83%, respectively), with moderate variation observed for these structural measurements within the cohort. To further explore the interrelationships among these traits, Pearson correlation analysis was conducted. The results showed strong positive and highly significant pairwise correlations across the four body measurement traits (r = 0.82–0.94, *p* < 0.001). The strongest correlation was observed between WH and HG (r = 0.94), while the weakest correlation was detected between BW and BL (r = 0.82) (Figure S1). Collectively, these findings demonstrate that the four body measurement traits are tightly interrelated and collectively reflect the overall growth and development of Hexi cattle, further supporting the reliability of subsequent GWAS results for these correlated traits.

### 3.2. Analysis of Population Genetic Structure

**Figure 1:**
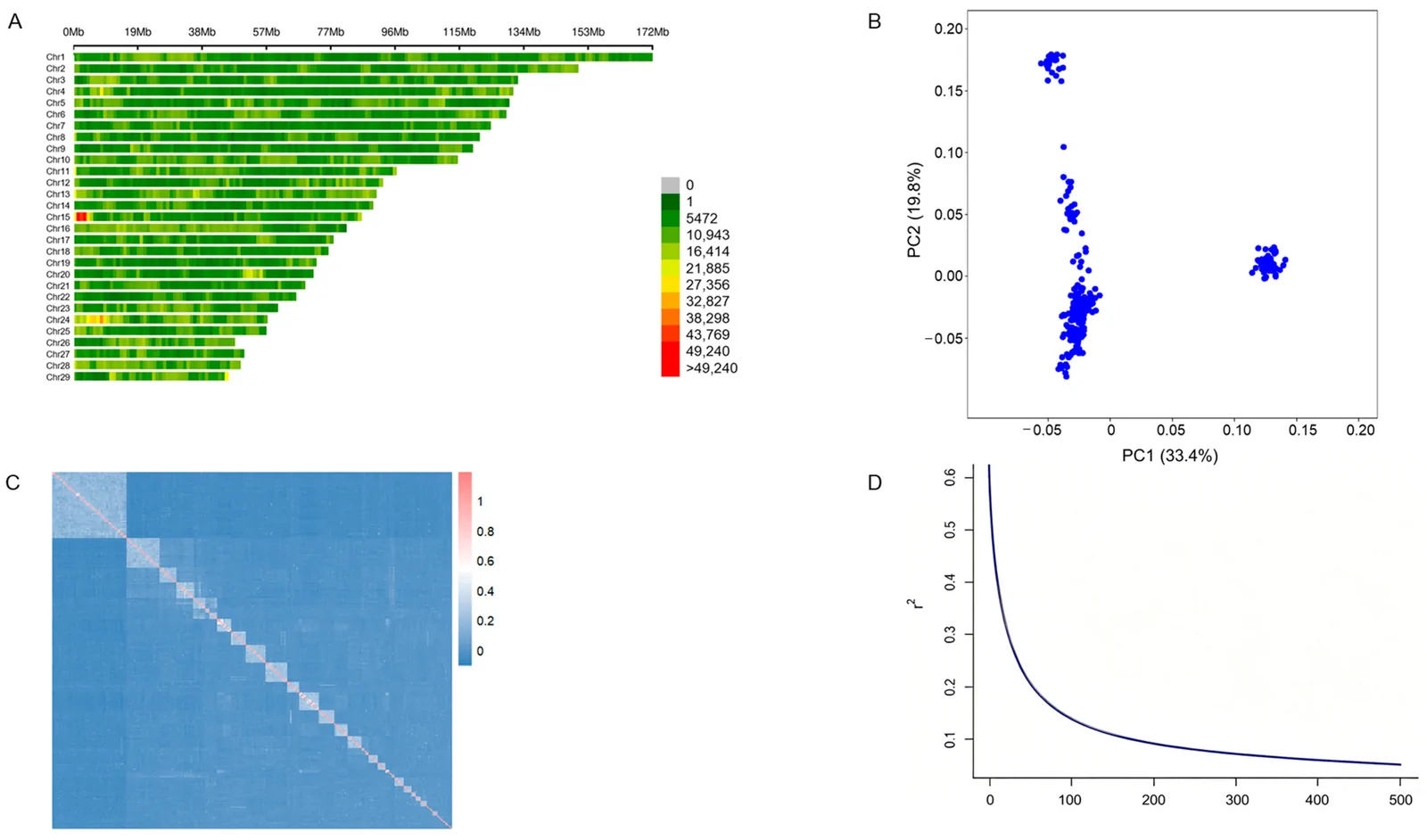
Population Genetic Structure and Linkage Disequilibrium Analysis in Hexi Cattle (**A**) Chromosomal Distribution of SNPs; (**B**) PCA plot; (**C**) Heatmap of Genomic Relationship Matrix; (**D**) LD decay plot.

Whole-genome resequencing was performed on 264 Hexi cattle, and high-quality sequencing data were obtained. All samples exhibited Q20 and Q30 values above 95%, a GC content ranging from 40% to 50%, and an average sequencing depth of 22.14× (Table S1). After quality control and sequence alignment, effective clean reads were evenly distributed across the 29 chromosomes of Hexi cattle (Figure 1A). Principal component analysis (PCA) based on genome-wide SNPs identified two distinct genetic clusters among individuals from the three breeding farms. Specifically, cattle from one farm formed an independent genetic cluster that was clearly differentiated from the other two populations, while individuals from the remaining two farms extensively overlapped without obvious separation. PC1 and PC2 explained 33.4% and 19.8% of the total genetic variance, respectively, indicating highly similar genetic backgrounds and consistent population genetic structures between these two groups (Figure 1B). Genomic relationship matrix (GRM) analysis revealed weak overall genetic relatedness across the Hexi cattle population. Nevertheless, several pairs of closely related individuals were identified, suggesting that the population does not conform to random mating and retains inherent familial pedigree structures (Figure 1C). LD analysis showed that the LD coefficient r^2^ decayed to 0.1 at a physical distance of approximately 70 kb. The r^2^ value declined steadily as the physical distance between SNP loci increased, demonstrating that closer chromosomal distances between SNPs correspond to stronger linkage disequilibrium (Figure 1D).

### 3.3. Genome-Wide Association Study of Body Weight and Measurement Traits

**Figure 2:**
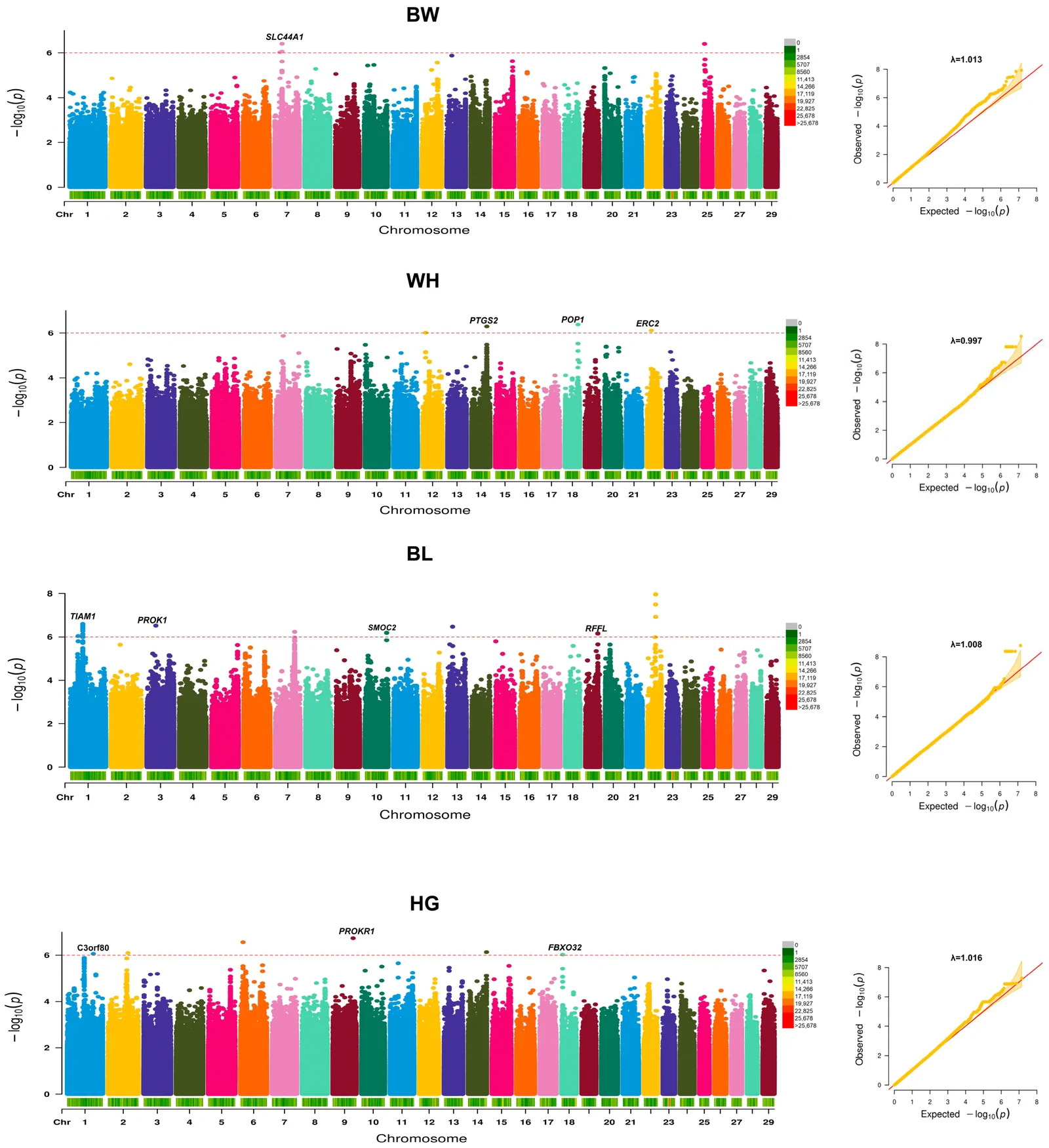
Manhattan and QQ plots of body measurement traits in Hexi cattle. The dashed horizontal line represents the genome-wide suggestive significance threshold. BW: Body weight; WH: withers height; BL: body length; HG: heart girth.

The genomic inflation factors (λ) for BW, WH, BL, and HG were 1.013, 0.997, 1.008, and 1.016, respectively. All observed λ values ranged from 0.997 to 1.016, indicating that population stratification was adequately corrected and supporting the validity of the GWAS results. A total of 11 candidate genes were identified through functional annotation of significantly associated SNPs. Three SNPs significantly associated with BW were only annotated to the *SLC44A1* gene (Figure 2A). Three SNPs significantly associated with WH were annotated to *PTGS2*, *POP1* and *ERC2* (Figure 2B), and nine SNPs linked to BL were annotated to *TIAM1*, *PROK1*, *SMOC2* and *RFFL* (Figure 2C). For HG, the annotated candidate genes included *C3orf80*, *PROKR1* and *FBXO32* (Figure 2D). Functional annotation of these significant SNPs was performed using ANNOVAR (version 2021-08-19). The results showed that none of the SNPs mapped to exonic regions; some variants were intronic, while others resided in the upstream or downstream regions of adjacent genes (Table S2). We calculated the phenotypic variance explained (PVE) for each significant SNP and the cumulative PVE of the 11 candidate genes. The PVE values of these significant SNPs ranged from 0.65% to 2.67% (Table S2).

### 3.4. Genotyping of Single-Nucleotide Polymorphism Loci

**Figure 3:**
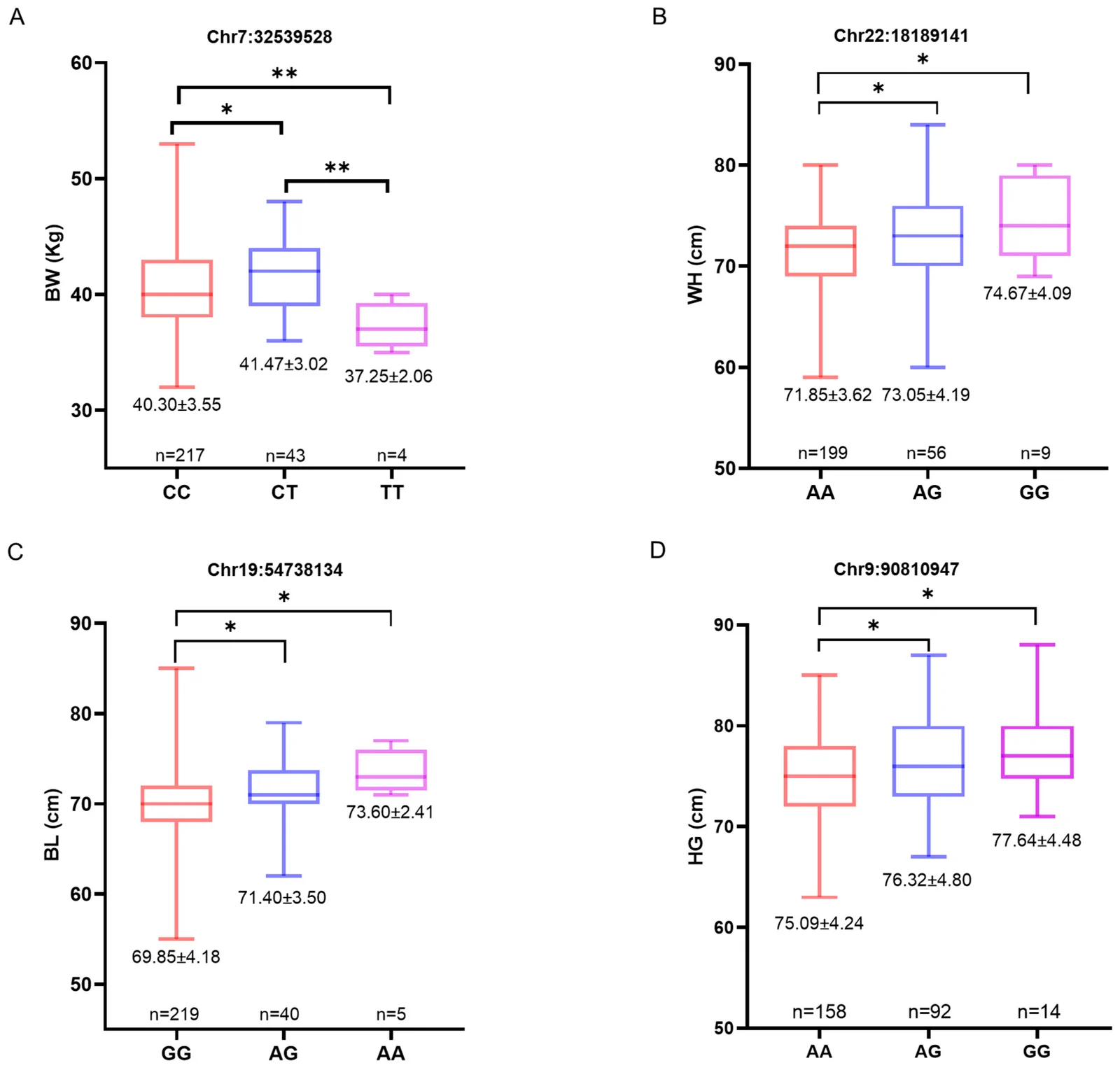
Genotyping results for key SNP loci in Hexi cattle. (**A**): Chr7:32539528; (**B**): Chr22:18189141; (**C**): Chr19:54738134; (**D**): Chr9:90810947. Significant relationships among the three genotypes. * indicates that 0.01 ≤ *p* < 0.05; ** indicates that *p* < 0.01.

Four significantly associated SNP loci (Chr7:32539528, Chr22:18189141, Chr19:54738134 and Chr9:90810947) were selected for visualization in this study. Based on physical proximity and functional annotation, each locus was sequentially annotated to the candidate genes *SLC44A1*, *ERC2*, *RFFL* and *PROKR1*, respectively. HWE test showed that all four loci conformed to Hardy–Weinberg equilibrium (*p* > 0.05) (Table S3). Genotyping analyses of the 264 Hexi cattle revealed distinct genotype–phenotype relationships at the four representative SNP loci. For BW, the heterozygous genotype exhibited the highest phenotypic value among the three genotypes; in contrast, for WH, BL, and HG, phenotypic values increased progressively from wild-type homozygotes to heterozygotes to mutant homozygotes, with wild-type homozygotes showing the lowest values and mutant homozygotes the highest values (*p* < 0.05) (Figure 3).

## 4. Discussion

The birth body measurement traits of Hexi cattle exhibited sexual dimorphism, with newborn males outperforming females in all measured traits, a well-established phenomenon in ruminants and other mammals [28,29,30,31]. From a genetic perspective, such sex-related phenotypic differences may arise from sex-linked loci, hormone-related pathways, and sex-specific regulatory effects on growth-related genes. Given the high conservation of genomes between sexes in mammals, these developmental differences are likely mediated, at least in part, by sex-specific adaptive mechanisms of the placenta [32]. Together, these findings provide the first comprehensive phenotypic baseline for body measurement traits at birth in Hexi cattle, laying a solid foundation for GWAS to dissect the underlying genetic architecture, prioritize candidate genes, and inform molecular breeding strategies.

In livestock production, birth weight and body measurement traits are economically important and serve as key phenotypic indicators for dissecting the genetic architecture of early growth through GWAS [33]. In this study, *SMOC2* was significantly associated with body length in Hexi cattle. This association may be linked to the biological function of *SMOC2*, a highly conserved extracellular matrix calcium-binding protein that regulates growth plate chondrocyte proliferation and differentiation, extracellular matrix remodeling, and bone formation via the Runx2 signaling pathway; this gene may participate in axial skeletal elongation and mammalian skeletal development [34]. Similarly, *PTGS2* was significantly associated with birth withers height. As a rate-limiting enzyme specifically upregulated during bovine embryogenesis, *PTGS2* modulates prostaglandin synthesis efficiency, thereby governing fetal endochondral ossification and skeletal development—this biological function may underpin the observed association with early growth performance [35,36,37]. *TIAM1* also showed a significant association with body length in Hexi cattle. Its involvement in actin cytoskeleton dynamics, promotion of mesenchymal osteogenic differentiation and proliferation, and maintenance of skeletal developmental homeostasis suggest a plausible biological basis for this association with bovine skeletal morphogenesis [38]. Furthermore, *PROK1* was also significantly associated with body length in Hexi cattle, potentially acting through the evolutionarily conserved PROK1–PROKR1 ligand–receptor axis, which activates downstream VEGF and FGF signaling to optimize the embryonic growth microenvironment, enhance fetal tissue differentiation and somatic development, and indirectly facilitate skeletal development by regulating embryonic angiogenesis and nutrient delivery; these functions may provide a potential biological context for the observed association [39,40].

The candidate genes associated with heart girth and birth weight are predominantly enriched in biological pathways potentially involved in muscle development and nutrient metabolism. Among the genome-wide significant loci identified for these traits, *SLC44A1* emerged as a top candidate significantly associated with birth weight in Hexi cattle. This gene encodes a choline transporter that may be critical for maintaining cellular metabolic homeostasis, which could provide essential substrates for tissue growth and development. In line with this metabolic role, *SLC44A1* has been independently annotated as a key growth- and metabolism-related gene in beef cattle breeds, including Angus and Hanwoo, and further exhibits strong signatures of artificial selection in beef cattle, as evidenced by integrative bovine multi-omics analyses [41]. Similarly, *FBXO32* was significantly associated with heart girth in Hexi cattle. As an E3 ubiquitin ligase, *FBXO32* could modulate skeletal muscle mass by targeting key proteins for proteasomal degradation during myogenesis. Notably, polymorphisms in this gene have been robustly linked to multiple growth traits in Nanyang cattle, including chest depth and body length, suggesting its potential utility as a functional molecular marker for somatic development in beef cattle [42]. Additionally, *PROKR1* also showed a significant association with heart girth. Mechanistically, *PROKR1* may activate the CREB–NR4A2 signaling axis, which drives myoblast proliferation and differentiation while promoting oxidative myofiber specification. These coordinated processes could collectively improve skeletal muscle development and potentially contribute to variations in thoracic girth in Hexi cattle [43].

The four genes identified in this study, namely *POP1*, *ERC2*, *RFFL*, and *C3orf80*, have not been previously associated with body weight or body measurement traits in cattle according to existing livestock genomics literature. Therefore, these are only putative positional candidate genes that may potentially contribute to growth-related phenotypes in Hexi cattle. Notably, our GWAS is the first to identify significant associations between genomic loci harbouring these genes and birth body measurements in Chinese cattle, implying their potential roles in early phenotypic development, possibly via poorly characterized developmental or metabolic pathways. However, such hypothesized biological functions remain unproven and should not be regarded as established. Nevertheless, the observed associations should be interpreted cautiously. Local linkage disequilibrium may create spurious association signals, where detected variants may merely tag the underlying causal mutations rather than exert direct biological effects. We cannot exclude the possibility that the true causal variants reside in adjacent genomic regions outside these four genes. Further validation, including independent replication in a separate Hexi cattle population and targeted functional assays, is required to clarify the precise biological functions and genetic regulatory mechanisms of these genes. Linkage disequilibrium decay analysis revealed that r^2^ declined to 0.1 at approximately 70 kb in the Hexi cattle population. This feature carries important practical implications for marker-assisted selection. Generally, r^2^ > 0.1 is considered the minimum threshold for a marker to provide informative signals for selection. The observed LD decay pattern suggests that detectable associations between marker variants and causal mutations are confined to a short physical distance. Accordingly, the lead SNPs identified in this GWAS can serve as effective markers for marker-assisted selection only when the true causal mutation resides within this ~70 kb genomic window. Outside this range, weakened linkage disequilibrium will compromise the reliability of selection. This result also indicates that high-density SNP panels are required to efficiently capture causal variants and facilitate genomic selection targeting birth body measurement traits in Hexi cattle.

Beyond the identification of these novel candidate genes, our findings advance the understanding of the molecular genetic basis of early growth and development in Hexi cattle, expand the genomic resource database for indigenous Chinese cattle breeds, and provide functionally informed candidate genes along with theoretical grounding for marker-assisted and genomic selection strategies in Hexi cattle breeding programs. Nevertheless, it is important to acknowledge several limitations of the present study. First, the sample size (*n* = 264) is relatively modest for a genome-wide association study, which may reduce the statistical power to detect SNPs with small effects, particularly those with low minor allele frequencies; indeed, for some significant loci, the homozygous mutant genotype was present in only a few individuals (e.g., *n* = 4), limiting the reliability of genotypic comparisons for such rare genotypes, although the stringent significance threshold (*p* < 1 × 10^−6^) and the low genomic inflation factors (λ = 0.997–1.016) indicate that the identified associations are robust and not inflated by population stratification. Second, maternal information, including dam parity, dam age, and gestation length, was not available in the present study and therefore could not be incorporated as covariates in the association model; although all animals were born in the same year and season, potential maternal effects on birth traits could not be directly assessed. Third, the present study relies exclusively on population-level GWAS for candidate gene identification and statistical genetic association analysis, without experimental validation at the cellular or organismal level. Future work should therefore increase cohort sample sizes and integrate multi-omics approaches, including bulk and single-cell transcriptomics, to comprehensively characterize the regulatory architecture of these key genes and empirically validate their functional contributions. Collectively, these efforts will not only facilitate the precision breeding of elite Hexi cattle germplasm but also provide a paradigm for integrating GWAS with functional genomics to dissect complex traits in indigenous livestock breeds.

## 5. Conclusions

In this study, GWAS was performed on body weight and body measurement traits (withers height, body length, and heart girth) at birth using whole-genome resequencing data from 264 Hexi cattle. A total of 11 significantly associated candidate genes were identified via the mixed linear model. Specifically, *SLC44A1* was significantly associated with body weight; *PTGS2*, *POP1* and *ERC2* were significantly associated with withers height; *TIAM1*, *PROK1*, *SMOC2* and *RFFL* were significantly associated with body length; *C3orf80*, *PROKR1* and *FBXO32* were significantly associated with heart girth. Collectively, these findings provide new insights into the molecular mechanisms underlying early growth and development of Hexi cattle and offer a theoretical basis and genetic resources for marker-assisted selection and genomic selection breeding. However, further functional studies are warranted to clarify the biological functions of these candidate genes and their specific roles in the early growth and development of Hexi cattle.

## Figures and Tables

**Table 1:** Phenotypic values of body measurement traits in Hexi cattle.

Items	Sex (*n*)	Mean ± SD	Coefficient of Variation (CV)	Max	Min
BW (kg)	Male (125)	41.37 ± 3.19 ^a^	7.70%	53	35
	Female (139)	39.61 ± 3.55 ^b^	8.97%	51	32
WH (cm)	Male (125)	73.31 ± 3.04 ^a^	4.14%	84	65
	Female (139)	71.21 ± 4.15 ^b^	5.83%	80	59
BL (cm)	Male (125)	72.10 ± 3.68 ^a^	5.11%	85	60
	Female (139)	68.41 ± 3.69 ^b^	5.39%	77	55
HG (cm)	Male (125)	77.26 ± 4.41 ^a^	5.71%	88	67
	Female (139)	74.19 ± 4.04 ^b^	5.45%	87	63

Note: BW = body weight; WH = withers height; BL = body length; HG = heart girth. ^a,b^ For each trait, values bearing different superscripts indicate significant differences between males and females at *p* < 0.05.

## Data Availability

The data presented in this study are available on request from the corresponding authors.

## Supplementary Materials

The following supporting information can be downloaded at: https://www.mdpi.com/article/10.3390/ani16193133/s1, Table S1: Quality metrics of raw and filtered sequencing data; Table S2: Significant SNPs and Annotated Candidate Genes Associated with Growth Traits in Hexi Cattle; Table S3: Population genetic parameters and HWE test of four SNPs in Hexi cattle; Figure S1: Pearson Correlation among Four Body Measurement Traits.
