# Supplementary material for: Genome-Wide Association Study of Body Measurement Traits at Birth in Cattle
Source: Animals (Basel). 2026 Oct 6;16(19):3133. doi: 10.3390/ani16193133 (PMC13663506; doi:10.3390/ani16193133)
Supplement: Supplementary file 1 [file animals-16-03133-s001.zip › animals-4568497-supplementary.pdf]

Table S1 Quality metrics of raw and filtered sequencing data

Sample	Raw Base (bp)	Clean Base (bp)	Effective Rate (%)	Q20 (%)	Q30 (%)	GC Content (%)
1	58,649,891,700	58,376,043,548	99.53	98.92	97.11	43.38
2	60,514,384,200	59,571,947,868	98.44	99.58	98.38	43.86
3	59,069,894,700	58,768,736,156	99.49	98.91	97.09	43.84
4	62,454,059,100	62,198,311,902	99.59	98.71	96.82	44.11
5	59,603,043,300	59,320,347,972	99.53	98.98	97.26	43.69
6	70,211,581,800	68,408,197,264	97.43	99.52	98.24	44.01
7	58,025,993,100	57,745,658,770	99.52	98.90	97.14	43.74
8	60,969,327,300	60,638,805,718	99.46	98.93	97.19	43.83
9	61,147,050,900	60,883,659,796	99.57	98.94	97.28	43.78
10	60,064,215,600	59,825,363,108	99.60	98.89	97.10	43.62
11	61,281,704,700	61,012,917,838	99.56	98.88	97.07	43.64
12	58,484,958,600	58,237,895,978	99.58	98.88	96.93	43.78
13	71,915,862,300	70,541,720,074	98.09	99.47	97.92	43.86
14	59,209,766,100	58,992,695,654	99.63	98.9	96.98	44.34
15	61,404,128,100	61,120,767,244	99.54	98.90	97.14	43.59
16	60,452,963,100	60,183,967,156	99.56	98.93	97.12	43.71
17	64,582,931,700	63,024,099,968	97.59	99.65	98.64	44.18
18	65,776,506,900	64,228,830,478	97.65	99.62	98.54	43.91
19	58,053,371,700	57,794,772,146	99.55	98.90	97.04	43.75
20	60,748,958,700	60,468,372,284	99.54	98.92	97.13	43.67
21	96,294,970,500	94,456,261,088	98.09	99.47	98.01	43.79
22	71,105,095,800	70,697,603,082	99.43	99.56	98.16	43.47
23	60,376,794,900	60,104,791,716	99.55	98.94	97.21	43.81
24	61,615,376,400	61,316,804,964	99.52	98.91	97.09	44.44
25	58,896,373,500	58,690,519,268	99.65	98.78	96.79	44.20
26	61,715,259,900	61,417,667,706	99.52	98.92	97.21	43.86
27	59,634,583,800	59,341,780,794	99.51	98.93	97.23	43.46
28	60,871,875,600	60,578,505,110	99.52	98.94	97.13	43.91
29	58,950,084,600	58,698,317,332	99.57	98.96	97.19	44.05
30	62,661,436,500	62,324,653,134	99.46	98.99	97.29	43.91
31	59,428,773,900	59,120,841,382	99.48	98.98	97.25	43.98
32	61,207,065,900	60,921,119,164	99.53	98.97	97.19	43.80
33	60,870,858,900	60,645,487,764	99.63	98.83	96.91	44.14
34	59,185,554,600	58,927,351,748	99.56	99.01	97.31	43.40
35	61,633,227,900	61,371,337,252	99.58	98.89	97.09	43.79
36	59,445,823,500	59,183,387,930	99.56	98.93	97.17	43.62
37	59,090,663,100	58,743,884,128	99.41	98.90	97.11	43.55
38	60,142,124,400	59,880,926,276	99.57	98.86	97.05	43.71
39	70,161,264,000	68,653,417,316	97.85	99.56	98.25	43.92
40	62,658,081,900	62,379,551,602	99.56	99.01	97.31	43.84
41	64,436,405,100	63,418,412,496	98.42	99.34	97.48	44.11
42	61,728,164,100	61,437,947,750	99.53	98.96	97.27	43.99
43	60,658,058,400	60,409,471,774	99.59	98.83	96.88	44.09
44	59,736,018,600	59,481,339,038	99.57	98.88	97.03	43.73
45	60,010,439,400	59,728,999,128	99.53	98.96	97.24	43.68
46	57,200,380,800	56,916,241,036	99.50	98.85	96.99	43.70

47	62,232,656,100	61,940,616,714	99.53	98.93	97.14	43.93
48	59,739,334,500	59,494,419,860	99.59	98.89	97.08	43.62
49	59,352,852,900	59,107,889,488	99.59	99.03	97.35	43.53
50	58,694,173,200	58,409,207,772	99.51	98.94	97.18	43.71
51	59,684,020,200	59,449,385,728	99.61	98.85	96.93	44.33
52	60,709,722,900	59,839,093,150	98.57	99.40	97.65	43.59
53	62,438,792,700	62,212,536,958	99.64	98.92	97.15	43.64
54	60,766,692,300	60,493,726,672	99.55	98.90	97.09	43.74
55	61,624,990,800	61,344,635,192	99.55	98.91	97.13	43.69
56	60,377,703,600	60,148,505,990	99.62	98.88	96.97	43.91
57	59,633,663,400	59,366,241,666	99.55	98.94	97.16	43.64
58	60,070,530,900	59,829,681,580	99.60	98.88	97.01	43.82
59	59,062,903,800	58,832,175,228	99.61	98.99	97.27	43.81
60	61,765,999,200	61,555,550,416	99.66	98.78	96.79	44.04
61	59,309,416,500	59,083,455,420	99.62	98.94	97.19	43.60
62	59,368,771,500	59,112,091,930	99.57	98.89	97.04	43.63
63	61,390,727,100	60,351,493,702	98.31	99.44	97.81	43.60
64	61,837,172,700	61,576,474,208	99.58	98.96	97.22	43.68
65	58,414,395,600	58,152,626,274	99.55	98.96	97.11	43.70
66	61,420,082,700	61,193,443,586	99.63	98.92	97.17	43.5
67	61,803,549,300	61,585,256,430	99.65	98.91	97.03	43.77
68	60,774,912,900	60,533,885,644	99.60	98.89	97.06	43.77
69	58,624,870,800	58,411,464,290	99.64	98.88	96.8	43.89
70	58,861,562,100	58,599,239,308	99.55	98.93	97.16	43.75
71	67,814,394,000	67,540,353,928	99.60	99.14	97.35	43.54
72	60,460,371,900	60,198,911,598	99.57	98.90	97.06	43.74
73	58,018,815,600	57,771,431,760	99.57	98.90	97.03	43.71
74	59,355,160,800	59,112,193,774	99.59	98.90	97.05	43.64
75	58,051,108,500	57,803,468,662	99.57	99.00	97.24	43.62
76	58,763,300,700	58,521,817,484	99.59	98.94	97.08	43.76
77	58,710,366,600	58,524,437,814	99.68	98.87	96.94	44.21
78	66,992,524,500	66,247,461,978	98.89	99.25	97.91	44.20
79	65,066,585,700	64,483,329,300	99.10	99.19	97.79	44.25
80	61,438,013,400	60,948,517,456	99.20	99.03	97.49	44.16
81	63,583,930,500	63,077,026,414	99.20	99.19	97.80	44.38
82	64,169,666,100	63,567,822,314	99.06	99.15	97.74	44.16
83	70,866,245,100	69,534,769,266	98.12	99.6	98.41	44.08
84	66,027,038,400	65,413,365,142	99.07	99.13	97.69	44.03
85	64,302,724,200	63,680,999,310	99.03	99.20	97.70	44.21
86	63,297,477,600	62,720,835,840	99.09	99.05	97.46	44.03
87	61,966,955,400	61,308,525,278	98.94	99.26	97.87	44.25
88	63,183,360,000	62,474,967,922	98.88	99.17	97.72	44.27
89	59,153,971,200	58,440,947,846	98.79	99.12	97.59	44.21
90	62,301,925,800	61,994,079,728	99.51	99.15	97.65	43.84
91	63,221,135,700	62,534,760,992	98.91	99.12	97.63	44.12
92	63,567,356,700	63,149,748,036	99.34	99.12	97.62	43.97
93	61,797,540,900	61,308,175,558	99.21	99.13	97.65	44.05
94	57,193,720,500	56,556,333,446	98.89	99.48	97.9	43.94

95	62,352,756,000	61,279,345,514	98.28	99.57	98.29	44.20
96	69,719,998,500	68,420,485,882	98.14	99.54	98.14	44.29
97	67,184,127,000	65,725,307,154	97.83	99.54	98.15	44.41
98	69,548,854,800	68,087,412,508	97.90	99.53	98.11	44.32
99	61,034,468,400	59,596,489,846	97.64	99.54	98.17	44.43
100	59,786,533,500	59,267,946,378	99.13	99.52	98.06	43.94
101	60,026,612,400	59,152,626,738	98.54	99.47	97.89	44.05
102	59,881,079,700	58,885,183,974	98.34	99.50	98.00	44.04
103	70,085,972,700	69,066,718,466	98.55	99.51	98.05	44.24
104	68,437,946,100	65,420,226,192	95.59	99.64	98.65	43.79
105	66,515,935,500	65,718,091,644	98.80	99.52	98.09	44.20
106	62,903,990,400	62,184,490,154	98.86	99.51	98.03	44.15
107	71,815,669,500	70,752,163,758	98.52	99.54	98.15	44.12
108	62,240,670,600	61,638,208,592	99.03	99.51	98.02	43.93
109	65,628,951,300	64,555,811,150	98.36	99.54	98.17	44.25
110	59,657,234,400	58,846,077,474	98.64	99.47	97.85	43.91
111	67,402,219,800	65,866,952,500	97.72	99.60	98.43	44.00
112	65,416,010,100	64,643,679,708	98.82	99.52	98.07	44.16
113	63,795,649,200	62,998,093,420	98.75	99.63	98.52	44.26
114	62,381,930,700	61,609,728,740	98.76	99.56	98.29	44.06
115	67,304,247,000	66,504,712,582	98.81	99.67	98.59	44.06
116	70,639,822,800	69,765,716,604	98.76	99.66	98.66	44.09
117	69,006,621,900	68,298,906,956	98.97	99.67	98.72	43.91
118	64,759,456,500	64,355,904,468	99.38	99.64	98.58	43.99
119	66,556,173,900	65,790,744,760	98.85	99.69	98.81	44.13
120	62,231,463,600	61,427,190,198	98.71	99.67	98.72	44.18
121	70,006,671,000	68,168,103,462	97.37	99.60	98.35	44.04
122	69,720,587,100	68,189,616,984	97.80	99.57	98.26	43.92
123	69,122,690,700	67,600,226,810	97.80	99.69	98.70	44.27
124	70,226,009,100	68,483,018,704	97.52	99.61	98.40	44.02
125	69,465,331,800	67,551,093,028	97.24	99.66	98.68	44.05
126	71,498,076,300	69,034,675,522	96.55	99.68	98.78	43.25
127	66,537,417,300	64,949,831,294	97.61	99.62	98.53	43.84
128	71,361,645,000	69,659,931,416	97.62	99.63	98.54	43.88
129	70,222,555,800	66,796,006,278	95.12	99.72	98.92	45.45
130	67,500,852,600	65,893,999,062	97.62	99.64	98.62	44.07
131	65,066,143,800	63,691,041,206	97.89	99.62	98.53	43.85
132	71,599,920,600	69,960,891,998	97.71	99.63	98.55	44.11
133	65,910,969,300	64,309,420,736	97.57	99.66	98.67	43.94
134	64,285,086,600	62,875,772,566	97.81	99.65	98.63	43.8
135	65,285,659,200	63,855,961,134	97.81	99.62	98.53	43.76
136	62,382,698,700	60,935,825,210	97.68	99.63	98.55	43.79
137	67,336,509,900	65,868,471,040	97.82	99.64	98.61	43.86
138	71,422,476,600	69,986,490,860	97.99	99.42	97.74	43.90
139	69,049,511,400	67,587,320,920	97.88	99.63	98.57	43.88
140	70,029,174,600	68,473,913,874	97.78	99.64	98.6	43.86
141	69,993,277,200	68,580,737,512	97.98	99.72	98.94	44.15
142	60,737,404,200	60,484,379,822	99.58	98.99	97.3	43.75

143	61,362,335,400	61,077,988,210	99.54	98.92	97.15	43.83
144	60,064,956,300	59,821,388,130	99.59	98.95	97.30	43.85
145	62,330,287,200	61,973,041,682	99.43	98.94	97.29	43.91
146	63,659,390,700	63,371,678,070	99.55	99.00	97.32	43.80
147	63,364,656,000	63,108,370,886	99.60	98.94	97.09	43.93
148	61,963,035,300	61,652,712,200	99.50	99.07	97.49	43.78
149	60,801,604,500	60,496,844,272	99.50	98.87	97.12	43.72
150	62,423,404,200	62,124,133,140	99.52	98.96	97.21	43.85
151	64,473,271,200	64,195,106,170	99.57	98.86	96.92	44.18
152	63,228,131,100	62,971,914,128	99.59	98.97	97.24	43.75
153	60,702,841,200	60,398,598,056	99.50	98.87	97.11	43.86
154	62,925,205,500	62,605,061,802	99.49	99.03	97.42	43.82
155	62,852,428,200	62,583,775,302	99.57	98.91	97.13	43.91
156	62,966,585,400	62,600,175,886	99.42	98.99	97.35	43.95
157	61,472,092,800	61,159,901,240	99.49	98.89	97.17	43.71
158	60,905,308,800	60,577,263,792	99.46	98.95	97.31	43.79
159	71,752,464,600	70,026,243,086	97.59	99.65	98.62	44.10
160	62,967,425,400	62,649,201,128	99.49	98.95	97.23	43.65
161	65,281,326,600	64,944,881,284	99.48	98.96	97.10	43.79
162	62,073,162,000	61,764,566,728	99.50	99.02	97.42	44
163	62,590,815,600	62,275,834,520	99.50	99.04	97.36	43.86
164	65,260,073,100	64,966,653,928	99.55	98.98	97.22	43.81
165	61,109,850,000	60,815,974,946	99.52	98.87	97.13	43.89
166	58,319,902,200	58,070,067,472	99.57	98.90	97.12	43.88
167	64,558,572,600	64,245,073,546	99.51	98.93	97.15	43.78
168	64,302,922,200	64,024,125,592	99.57	98.89	97.15	43.79
169	63,412,336,800	63,082,556,664	99.48	98.96	97.29	43.82
170	63,184,624,800	62,901,427,226	99.55	98.95	97.24	43.79
171	63,747,480,600	63,400,891,590	99.46	99.06	97.48	43.9
172	61,061,962,500	60,754,479,086	99.50	98.86	97.07	43.91
173	62,958,458,100	62,677,190,102	99.55	98.86	97.10	43.85
174	63,166,167,300	62,896,855,954	99.57	98.88	96.98	43.77
175	61,735,142,100	61,493,431,684	99.61	98.92	97.15	43.95
176	61,992,704,100	61,690,339,456	99.51	98.92	97.14	43.69
177	61,105,721,100	60,855,204,074	99.59	98.87	96.99	43.85
178	60,149,390,400	59,856,398,752	99.51	99.01	97.31	43.97
179	62,566,364,700	62,207,063,890	99.43	99.06	97.43	43.92
180	60,318,245,400	60,074,595,060	99.60	98.90	97.02	43.76
181	61,854,280,200	61,568,245,112	99.54	98.93	97.24	43.92
182	63,240,003,000	62,983,564,612	99.59	98.95	97.20	43.74
183	61,518,564,000	61,278,007,880	99.61	98.91	97.1	43.97
184	63,662,861,100	63,377,016,694	99.55	99.02	97.35	43.81
185	60,708,956,400	60,493,857,272	99.65	98.94	97.13	43.9
186	60,641,763,300	60,364,075,880	99.54	98.99	97.30	43.94
187	59,216,573,400	58,975,740,098	99.59	98.98	97.26	43.76
188	58,131,010,800	57,898,305,670	99.60	98.93	97.03	43.95
189	61,101,771,600	60,803,932,762	99.51	98.86	97.05	43.89
190	61,155,364,200	60,878,735,186	99.55	99.02	97.36	43.76

191	57,884,257,500	57,666,550,858	99.62	98.89	97.09	43.87
192	59,753,047,800	59,487,198,850	99.56	98.96	97.26	43.86
193	66,565,901,100	66,245,814,484	99.52	98.97	97.13	43.72
194	68,816,699,100	67,011,224,432	97.38	99.58	98.35	44.09
195	64,075,640,100	63,802,668,332	99.57	99.03	97.37	44.04
196	59,590,059,900	59,347,714,700	99.59	98.95	97.20	43.94
197	60,949,047,600	60,725,282,710	99.63	98.98	97.32	43.99
198	60,382,097,400	60,077,942,912	99.50	98.97	97.21	43.79
199	61,311,936,300	61,047,526,522	99.57	98.96	97.25	43.69
200	62,958,462,300	62,715,078,356	99.61	99.02	97.17	43.89
201	61,365,060,900	61,086,129,334	99.55	98.94	97.16	43.73
202	61,389,837,300	61,132,820,316	99.58	98.94	97.19	43.78
203	70,149,857,100	69,823,459,654	99.53	98.96	97.09	43.67
204	71,791,293,600	71,441,387,914	99.51	99.13	97.53	43.94
205	69,136,369,500	68,822,242,570	99.55	98.97	97.19	43.81
206	62,376,430,500	62,035,006,644	99.45	98.94	97.24	43.91
207	60,815,573,100	60,515,795,390	99.51	98.85	97.01	43.73
208	57,660,771,600	57,330,099,096	99.43	98.94	97.17	44.16
209	71,452,694,700	71,061,336,228	99.45	98.97	97.01	43.88
210	61,247,541,300	60,962,239,422	99.53	98.89	97.11	43.86
211	57,258,057,600	57,011,375,974	99.57	98.89	97.12	43.77
212	57,695,709,000	57,427,116,368	99.53	98.95	97.12	43.63
213	58,954,216,200	58,669,624,556	99.52	98.85	96.91	43.67
214	70,297,107,900	68,826,858,672	97.91	99.65	98.63	43.97
215	60,843,445,200	60,551,014,808	99.52	98.72	96.65	44.57
216	62,643,875,700	62,290,003,132	99.44	99.07	97.45	43.84
217	61,225,946,100	60,942,283,292	99.54	98.91	97.17	43.93
218	61,273,197,000	60,954,834,548	99.48	98.92	97.20	43.71
219	61,824,031,800	61,537,076,074	99.54	98.94	97.20	43.85
220	59,043,966,300	58,805,145,154	99.60	98.93	97.22	43.84
221	58,334,044,200	58,055,487,670	99.52	98.99	97.29	43.71
222	58,095,628,800	57,803,500,570	99.50	98.93	97.18	43.74
223	58,430,805,600	57,486,479,692	98.38	99.61	98.49	43.78
224	61,848,432,300	61,544,807,684	99.51	98.9	97.15	43.81
225	59,306,499,600	59,034,385,012	99.54	98.99	97.25	43.96
226	60,284,742,300	59,999,274,264	99.53	99.04	97.37	43.78
227	59,080,184,700	58,841,909,466	99.60	98.98	97.29	43.66
228	59,554,869,900	59,265,438,742	99.51	98.88	97.06	43.91
229	59,256,714,300	58,993,991,482	99.56	98.86	97.02	43.72
230	59,927,856,600	59,699,176,906	99.62	99.06	97.39	43.82
231	61,745,646,300	61,486,380,534	99.58	98.93	97.17	43.55
232	60,893,450,100	60,625,348,424	99.56	98.89	97.14	43.86
233	61,378,649,100	61,108,854,080	99.56	98.91	97.06	44.17
234	61,329,888,000	61,034,211,352	99.52	98.92	97.15	43.65
235	62,888,165,100	62,632,407,818	99.59	98.88	97.01	43.61
236	62,670,107,700	62,371,907,156	99.52	98.95	97.18	43.77
237	59,856,576,000	59,602,398,174	99.58	98.93	97.18	43.92
238	61,828,222,500	61,507,163,722	99.48	98.94	97.19	44.01

239	64,104,506,100	63,792,915,136	99.51	98.98	97.28	43.72
240	58,961,336,400	58,697,773,316	99.55	98.85	97.04	43.75
241	58,627,768,800	58,384,488,778	99.59	98.99	97.31	43.51
242	59,536,380,900	59,264,142,286	99.54	98.94	97.22	43.72
243	57,602,571,600	57,429,829,422	99.70	99.05	97.39	43.8
244	61,169,361,300	60,918,889,514	99.59	98.97	97.26	43.64
245	60,930,448,200	60,712,345,206	99.64	98.81	96.78	44.03
246	63,165,624,600	62,905,420,370	99.59	98.99	97.30	43.71
247	61,129,119,600	60,853,801,638	99.55	98.93	97.20	43.71
248	59,140,363,200	58,730,490,478	99.31	98.83	96.96	43.76
249	60,554,399,100	60,246,474,084	99.49	99.00	97.33	43.82
250	60,143,239,200	59,873,323,278	99.55	98.94	97.14	44.02
251	68,564,042,700	68,242,683,382	99.53	99.04	97.31	44.01
252	62,339,082,000	62,066,483,072	99.56	98.96	97.18	43.43
253	61,971,246,600	61,679,607,578	99.53	98.96	97.23	43.76
254	62,454,782,400	62,160,195,724	99.53	98.93	97.16	43.92
255	60,756,230,100	60,510,191,698	99.60	98.98	97.30	43.67
256	60,841,217,700	60,603,949,294	99.61	98.94	97.21	43.77
257	65,485,016,700	65,170,300,828	99.52	99.06	97.43	43.83
258	57,524,817,000	57,293,763,602	99.60	99.01	97.33	43.75
259	61,621,001,100	61,344,683,350	99.55	98.9	97.05	44.28
260	58,889,509,500	58,627,185,602	99.55	98.98	97.26	43.54
261	61,601,649,900	61,232,480,696	99.40	98.98	97.41	43.73
262	57,174,363,000	56,890,148,352	99.50	98.85	97.06	43.82
263	59,968,530,600	59,709,290,988	99.57	98.97	97.24	43.73
264	59,407,106,700	59,093,979,144	99.47	99.59	98.31	44.26

Table S2 Significant SNPs and Annotated Candidate Genes Associated with Growth Traits in Hexi Cattle

Position	Trait	Ref/Alt	MAF	SNP effect	Standard Error	*P*	Candidate gene	PVE (%)	Variant annotation
Chr7:32539484	BW	A/T	0.12	1.11	0.22	7.59E-07	*SLC44A1*	2.10	upstream
Chr7:32539507	BW	T/A	0.34	0.78	0.16	6.31E-07	*SLC44A1*	2.26	upstream
Chr7:32539528	BW	C/T	0.10	1.01	0.20	7.31E-07	*SLC44A1*	1.45	upstream
Chr14:67809151	WH	A/T	0.21	1.08	0.22	6.03E-07	*PTGS2*	2.67	intron
Chr18:58000365	WH	G/A	0.15	0.77	0.15	4.17E-07	*POP1*	1.02	upstream
Chr22:18189141	WH	A/G	0.10	1.17	0.24	8.51E-07	*ERC2*	1.63	intron
Chr1:34397919	BL	A/G	0.22	0.95	0.18	2.24E-07	*TIAM1*	1.82	downstream
Chr1:34397752	BL	T/G	0.19	0.94	0.19	7.08E-07	*TIAM1*	1.59	downstream
Chr1:34397831	BL	A/G	0.20	0.94	0.19	4.79E-07	*TIAM1*	1.68	downstream
Chr1:34398014	BL	G/A	0.15	0.90	0.18	8.32E-07	*TIAM1*	1.25	downstream
Chr1:34398156	BL	G/A	0.24	0.95	0.19	5.62E-07	*TIAM1*	1.91	downstream
Chr1:34398289	BL	G/A	0.18	0.91	0.18	7.76E-07	*TIAM1*	1.43	downstream
Chr3:39618244	BL	C/T	0.24	0.64	0.13	5.01E-07	*PROK1*	0.88	downstream
Chr10:96765344	BL	G/T	0.20	0.80	0.17	9.33E-07	*SMOC2*	2.81	downstream
Chr19:54738134	BL	G/A	0.12	0.83	0.17	9.12E-07	*RFFL*	1.93	upstream
Chr1:116723413	HG	T/C	0.20	0.73	0.15	8.91E-07	*C3orf80*	0.84	upstream
Chr9: 90810947	HG	A/G	0.15	1.15	0.23	3.16E-07	*PROKR1*	1.65	upstream
Chr18: 3921390	HG	A/G	0.12	0.79	0.16	6.31E-07	*FBXO32*	0.65	intron

Notes: BW, birth weight; WH, withers height; BL, body length; HG, heart girth; MAF, minor allele frequency; PVE, proportion of phenotypic variance explained by each SNP.

Table S3 Population-genetic parameters and HWE test of four SNPs in Hexi cattle

SNPs	Genotypic Frequencies			Allelic Frequencies		Ho	He	PIC	*p* Value
g. 32539528 C>T	CC (217)<br>0.822	CT (43)<br>0.163	TT (4)<br>0.015	C (477)<br>0.903	T (51)<br>0.097	0.825	0.175	0.159	0.318
g. 18189141 A>G	AA (199)<br>0.754	AG (56)<br>0.212	GG (9)<br>0.034	A (454)<br>0.860	G (74)<br>0.140	0.758	0.241	0.219	0.405
g. 54738134 G>A	GG (219)<br>0.829	AG (40)<br>0.152	AA (5)<br>0.019	G (478)<br>0.905	A (50)<br>0.095	0.829	0.171	0.157	0.313
g. 90810947 A>G	AA (158)<br>0.599	AG (92)<br>0.348	GG(14)<br>0.053	A (408)<br>0.773	G (120)<br>0.227	0.649	0.351	0.290	0.536

*Note: Ho: homozygosity; He: heterozygosity; polymorphism information content (PIC) values less than 0.25 indicate low polymorphism, values between 0.25 and 0.5 indicate moderate polymorphism, and values greater than 0.5 indicate high polymorphism. A *p* value > 0.05 indicates that the population's genes are in Hardy–Weinberg equilibrium, suggesting that the samples originated from the same Mendelian population.

Figure S1 Pearson Correlation among Four Body Measurement Traits

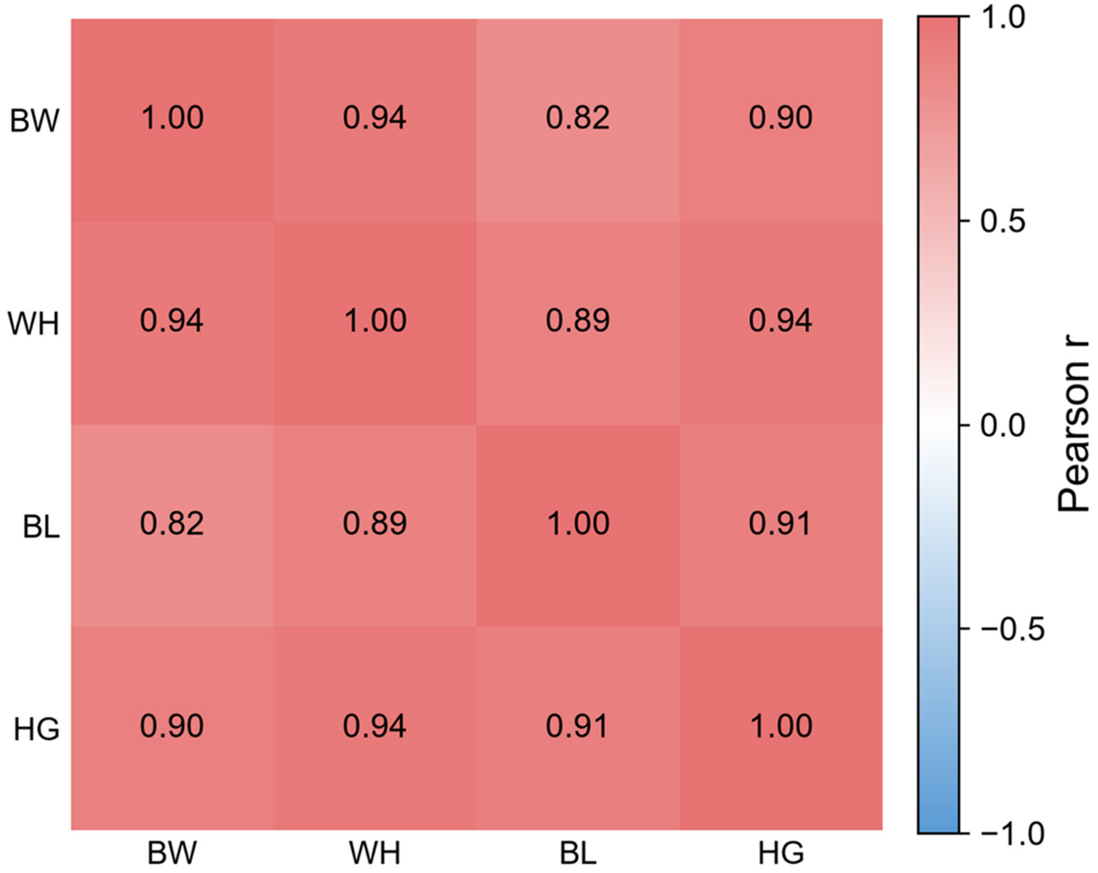

Notes: BW, birth weight; WH, withers height; BL, body length; HG, heart girth.
